# Prediction of cancer survivors’ mortality risk in Korea: a 25-year nationwide prospective cohort study

**DOI:** 10.4178/epih.e2022075

**Published:** 2022-09-13

**Authors:** Yeun Soo Yang, Heejin Kimm, Keum Ji Jung, Seulji Moon, Sunmi Lee, Sun Ha Jee

**Affiliations:** 1 Department of Public Health, Yonsei University Graduate School, Seoul, Korea; 2 Department of Epidemiology and Health Promotion, Institute for Health Promotion, Graduate School of Public Health, Yonsei University, Seoul, Korea; 3 Health Insurance Policy Research Institute, National Health Insurance Service, Wonju, Korea

**Keywords:** Cancer survivor, Prediction, Mortality risk factors, Life style, Tobacco smoking

## Abstract

**OBJECTIVES:**

This study aimed to investigate the factors affecting cancer survival and develop a mortality prediction model for Korean cancer survivors. Our study identified lifestyle and mortality risk factors and attempted to determine whether health-promoting lifestyles affect mortality.

**METHODS:**

Among the 1,637,287 participants in the Korean Cancer Prevention Study (KCPS) cohort, 200,834 cancer survivors who were alive after cancer diagnosis were analyzed. Discrimination and calibration for predicting the 10-year mortality risk were evaluated. A prediction model was derived using the Cox model coefficients, mean risk factor values, and mean mortality from the cancer survivors in the KCPS cohort.

**RESULTS:**

During the 21.6-year follow-up, the all-cause mortality rates of cancer survivors were 57.2% and 39.4% in men and women, respectively. Men, older age, current smoking, and a history of diabetes were high-risk factors for mortality, while exercise habits and a family history of cancer were associated with reduced risk. The prediction model discrimination in the validation dataset for both KCPS all-cause mortality and KCPS cancer mortality was shown by C-statistics of 0.69 and 0.68, respectively. Based on the constructed prediction models, when we modified exercise status and smoking status, as modifiable factors, the cancer survivors’ risk of mortality decreased linearly.

**CONCLUSIONS:**

A mortality prediction model for cancer survivors was developed that may be helpful in supporting a healthy life. Lifestyle modifications in cancer survivors may affect their risk of mortality in the future.

## INTRODUCTION

Cancer is the leading cause of mortality worldwide, and the number of cancer survivors continues to increase in high-income and middle-income countries [1]. According to the 2018 Korea Central Cancer Registry, the 5-year relative survival rate in patients with cancer who were diagnosed in a recent 5-year period (2014–2018) was 70.3%, which means that 7 patients out of 10 patients survived for more than 5 years. This is 1.3 times higher (a 16.2 percentage point increase) than the 5-year survival rate of patients diagnosed with cancer 10 years previously (2001–2005) (54.1%) [2]. The Organization for Economic Cooperation and Development Health Care Quality Indicators for Cancer Care reported that the 5-year survival rates of breast cancer in the United States, Australia, Japan, and the United Kingdom were 90.2%, 89.5%, 89.4%, and 85.6%, respectively, in 2010–2014. The survival rates of cervical cancer in Denmark, Japan, the United Kingdom, and Korea in the same period were 69.5%, 71.4%, 63.8%, and 86.6%, respectively. The 5-year survival rates of colon cancer were 60.0%, 70.6%, 67.9%, and 71.8% in the United Kingdom, Australia, Belgium, and Korea, respectively. Although there are differences among cancer sites and countries, cancer survival rates are high and continuously increasing.

Several factors that contribute to cancer incidence are related to lifestyle, including lack of exercise, smoking, inappropriate diet, drinking, and obesity [3]. As is already known, smoking, drinking, and excess body weight increase cancer incidence and mortality [1,4–6], while high levels of physical exercise are associated with a lower cancer death rate [3,7]. Cancer survival is considered a key measure of the effectiveness of cancer services, capturing both how good the system is at detecting the disease and whether people have rapid access to effective treatment. However, the cancer survival rate can be changed not only through these systemic aspects, but also through individual changes and lifestyle management.

There is a lack of predictive studies targeting cancer survivors or studies using lifestyle as a predictor variable. In a predictive study of cancer survivors, Parikh et al. [8] observed 1,065 patients for 500 days and predicted the short-term mortality risk of patients with cancer within 6 months using age, sex, comorbidities, and laboratory values as predictors. Koczwara et al. [9] conducted a study to verify the cardiometabolic predictors of mortality in cancer survivors. A Dutch study also predicted mortality in elderly patients aged ≥65 years with breast cancer. Age, tumor size, tumor grade, nodal status, hormone receptor status, human epidermal growth factor receptor 2 status, and number of comorbidities were used as predictors [10]. Baek et al. [11] conducted a study on the prediction of late breast cancer-specific mortality in recurrence-free breast cancer survivors, and Gupta et al. [12] found that clinical outcomes could be predicted using machine learning applied to information from both a database dedicated to disease (in this case, cancer) and electronic administrative records.

Cancer survivors have several concerns about physical, practical, and emotional problems, but they do not receive appropriate information to help manage these concerns [13]. In addition, in the context of increasing cancer survival, there is a lack of studies on what should be managed to improve quality of life or survival, and there is also a lack of studies that predict mortality in cancer survivors in Asians considering these lifestyle factors as predictors or based on a large cohort that has been tracked for a long time. This study aimed to investigate the factors affecting mortality among cancer survivors and to build a mortality prediction model for cancer survivors based on these factors. Moreover, this study provides evidence that cancer survivors should manage their lifestyles appropriately.

## MATERIALS AND METHODS

### Study population

We used prospective cohort study data from the Korean Cancer Prevention Study (KCPS). The KCPS cohort includes the insured, who were government employees and private school staff, who were enrolled in the Korean Medical Insurance Corporation (currently the National Health Insurance Service [NHIS] as Government Employees’ Union and Private School Staff Union), and who underwent regular physical examinations at least once between 1992 and 1995 [14,15]. The KCPS cohort includes 1,637,287 Koreans, of whom 989,744 and 647,543 are men and women, respectively. This study included patients aged between 20 years and 95 years who had undergone 1 biennial medical evaluation using the NHIS. Of the 1,637,287 participants, 200,834 were cancer survivors (136,000 and 64,834 men and women, respectively).

In order to establish a prediction model for all-cause mortality and cancer death mortality of cancer survivors, 25 participants who reported having cancer at, or prior to, the initial visit were excluded. Furthermore, 1,821 participants with missing information on exercise status, body mass index (BMI), or who had an extremely high (>100 kg/m^2^) or low BMI (<16 kg/m^2^) were excluded. The final study participants were 198,988 individuals (Supplementary Material 1).

### Cancer survivors

Many cancer centers construct survival standards, initiatives, and survival treatment plans to address the requirements of patients who have completed their treatment. The focus of these therapy plans and programs is on those who have finished treatment and are deemed cured. However, because the demands of individuals with advanced cancer are frequently vastly distinct [16,17], there is a significant care gap for individuals who are currently living with cancer, as opposed to those who are living after cancer or dying from cancer.

The term “cancer survivor” was coined and first used in the United States for advocacy purposes and to encourage research and treatment for this growing demographic. Some organizations (e.g., the Office of Cancer Survivorship at the National Cancer Institute, the Centers for Disease Control and Prevention and the National Coalition for Cancer Survivorship) identify cancer survivors based on the date of the initial cancer diagnosis [16,18]. A recent review of the history, meaning, and subtleties of the terms “survivor” and “survivorship” suggested that the description “someone who has experienced cancer” may be better because it refers to all cancer patients and tacitly acknowledges their heterogeneity [19,20]. The purpose of this study was to provide necessary information to all cancer patients living with cancer or having been treated for cancer, and accordingly, a broad definition of cancer survivorship was used. Therefore, we made no distinction between “survivor” and “patient” and used “survivor” for both concepts. Our choice adheres to Mullan’s (1985) “seasons of survival” theory and the current definition of the National Coalition for Cancer Survivorship [16,18,21,22]. To summarize, we selected a definition of “cancer survivor” that refers to the individual from the moment of cancer diagnosis until the end of life.

### Data collection

The participants were instructed to self-report their lifestyle, including a history of smoking (never, former, current), alcohol consumption (g/day of ethanol), exercise participation (yes, no), and medical history, including hypertension (yes, no), diabetes (yes, no), and family medical history (yes, no). Height and weight were measured directly in light clothing with shoes removed. BMI was calculated as weight in kilograms divided by the square of height in meters. We classified BMI based on the World Health Organization Asian-Pacific classification, where adults with a BMI below 18.5 kg/m^2^ and between 18.5 and 22.9 kg/m^2^ are classified as “underweight” and “normal,” respectively. Above the normal range, there are conventional grades for “overweight” (23.0 kg/m^2^ ≤BMI<25.0 kg/m^2^) and “obese” (BMI≥25.0 kg/m^2^) individuals [23]. Blood pressure was measured while the participants remained seated using a standard mercury sphygmomanometer or an automatic manometer [14,24].

### Follow-up and outcomes

The baseline period of the KCPS was 1992 to 1995 for the development of a prediction model of 10-year all-cause mortality risk and 10-year cancer mortality risk. Our study participants were followed up until December 31, 2019. The mean follow-up periods of the general population and cancer survivors were 24.7 years and 21.6 years, respectively. The average survival period of cancer survivors following a cancer diagnosis was 6.6 years (6.0 years for men and 7.7 years for women). A cancer survivor was defined as a participant who survived after a diagnosis of cancer according to the National Coalition of Cancer Survivorship definition in this study [16,18,21,22,25]. In our study, cancer survivors accounted for 12.3% (n=200,834) of the 1,637,287 participants.

The outcomes included cancer-related mortality and all-cause mortality. Cancer death and all-cause mortality were evaluated based on certificates from the National Statistical Office, and abstractors coded the causes of cancer death and all-cause mortality using the International Classification of Disease, 10th edition (ICD-10). All-cause mortality follow-up started at the enrollment date and ended at death, and for cancer deaths, follow-up ended at deaths for which the ICD-10 code was cancer only.

### Statistical analysis

We calculated the cumulative cancer incidence and crude death rate per 100,000 person-years for all-cause mortality and cancer death in cancer survivors. To build a mortality prediction model for cancer survivors, discrimination and calibration in predicting the 10-year mortality risk and mortality risk factors in the KCPS were evaluated. The discriminatory power of the KCPS all-cause mortality risk (KAR) and KCPS cancer mortality risk (KCR) models in predicting mortality endpoints for the KCPS participants was assessed using the area under the receiver operating characteristic curve (AUC) or C-statistic [26]. Calibration analysis, which is a measure of how close the predicted risk is to the actual risk, was performed by dividing participants within cancer survivors into deciles of predicted risk. The observed and predicted 10-year mortality risks (all-cause and cancer deaths) in each decile were compared using the Hosmer–Lemeshow test. Calibration was also determined graphically by plotting the observed and predicted mortality events, grouped according to deciles of the predicted probability [27]. For KAR and KCR, the coefficients in the KCPS Cox proportional hazard models, mean values of the risk factors, and mean incidence rates in the KCPS cohort study were used. We divided the cohort data into 2 groups: a 50% random sampling of the derivation dataset for model derivation and the remaining 50% test dataset for model internal validation in cancer survivors (Supplementary Material 2). All analyses were performed using SAS version 9.4 (SAS Institute Inc., Cary, NC, USA).

### Ethics statement

This study was approved by the Institutional Review Board of Yonsei University (IRB No. 4-2001-0029).

## RESULTS

The number of cancer survivors was 200,834 (12.3%) from a total of 1,637,287 study participants from the KCPS. The mean age was higher in cancer survivors (48.9 years) than in the general population (42.9 years). The older age group of cancer survivors showed a higher cancer prevalence rate and higher rates of hypertension and diabetes history than the general population. The trend was similar in patients who had a family history of cancer (Table 1).

Of the 1,637,287 participants, 200,834 were diagnosed with cancer. A total of 103,320 (51.5%) of those 200,834 participants died, and 84.1% (86,891) of them died from cancer. The cumulative incidence was 497 per 100,000 individuals in the general population. When analyzed by gender, the incidence of cancer per 100,000 person-years in men and women was 559 and 402, respectively. The all-cause mortality from cancer survivors was 2,385 per 100,000 person-years, and the cancer death rate was 2,006 per 100,000 person-years (Table 2).

After stratification of the causes of death by specific cancer site, stomach cancer showed the highest incidence (39,926 individuals). The mortality rate from cancer was high in patients with lung, stomach, and liver cancers (Table 3). All-cause mortality was calculated as the sum of all cancer deaths and other deaths.

### Risk factors for cancer incidence and cancer mortality

Regarding the risk factors for mortality in cancer survivors, the cancer mortality rate was 1.05 higher (hazard ratio [HR], 1.05; 95% confidence interval [CI], 1.05 to 1.05) in the older group, and the risk was lower in participants who were overweight or obese than in their lower-BMI counterparts (HR, 0.99; 95% CI, 0.99 to 0.99). Current smokers showed a higher cancer mortality rate than non-smokers (HR, 1.44; 95% CI, 1.42 to 1.47), and participants with a history of diabetes showed a higher cancer mortality rate than those without diabetes (HR, 1.09; 95% CI, 1.06 to 1.13). The participants who exercised also showed a lower cancer mortality rate (HR, 0.97; 95% CI, 0.96 to 0.98) than those who did not exercise. Patients with a family history of cancer (HR, 0.93; 95% CI, 0.91 to 0.95) and history of hypertension (HR, 0.93; 95% CI, 0.90 to 0.96) showed protective effects against cancer mortality (Table 4). To control for non-linearity in BMI, it was stratified into 4 groups and analyzed using a Cox proportional hazards model. The results were similar, with the normal-weight (18.5–22.9 kg/m^2^), overweight (23.0≤BMI<25.0 kg/m^2^), and obese (BMI≥25.0 kg/m^2^) BMI groups showing lower mortality rates than the underweight group (less than 18.5 kg/m^2^) (Supplementary Material 3).

### Cancer survivors’ mortality prediction model

The dataset was randomly divided into derivation (n=99,489) and validation (n=99,499) sets based on 50% of the participants, and the mortality prediction model was set using 10-year risk and regression coefficients (Supplementary Material 2). We estimated the 10-year risk of mortality in cancer survivors since the average survival period following a cancer diagnosis was 6.6 years (6.0 years for men [standard deviation (SD), 5.8] and 7.7 years for women [SD, 6.1]).

### Calibration for 10-year all-cause mortality prediction from cancer survivors

Calibration refers to how close the predicted risk of disease is to the actual observed risk. This study calculated the KAR and KCR models using Cox coefficients, and the averages of each variable were calculated for age, gender, BMI, smoking (ex-smoking and current smoking), exercise, family history of cancer, and medical history of hypertension and diabetes. The predicted and observed actual risks were compared by dividing them into deciles.

The equation for the all-cause mortality prediction model was as follows:

$$\begin{array}{l} \text{KAR}=0.05869\times (\text{AGE}-48.7764)-0.21819\times (\text{GENDER}-1.32291)-\\ 0.00961\times (\text{BMI}-23.211)+0.06191\times (\text{EXSMOK}-0.146)+0.35342\times \\ (\text{CUSMOK}-0.43309)-0.04934\times (\text{EXER}-1.26239)-0.09434\times (\text{FCAN}-\\ 0.26239)-0.04467\times (\text{PHTN}-0.049352)+0.17932\times (\text{PDM}-0.0352);\\ \text{KAR}1=\text{exp}(\text{KAR}); \end{array}$$

BMI, body mass index; EXSMOK, former smoker; CUSMOK, current smoker; EXER, exercise; FCAN, family history of cancer; PHTN, past history of hypertension; PDM, past history of diabetes.

The predicted score for cancer survivors was calculated as 1− 0.53203×KAR1, reflecting the 10-year survival rate.

**Figure 1:**
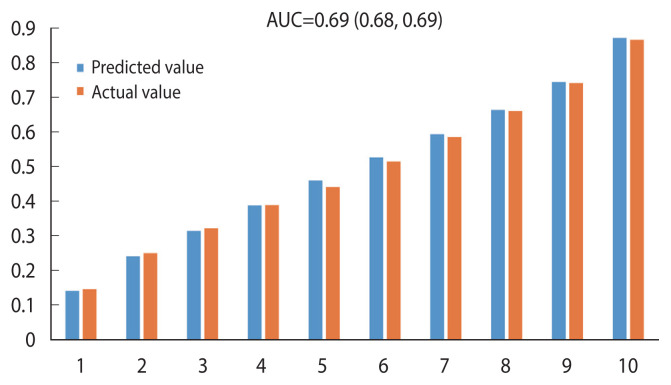
Calibration for 10-year all-cause mortality prediction in cancer survivors. AUC, area under the receiver operating charac teristic curve.

Based on the 10-year all-cause mortality prediction model for cancer survivors, the Hosmer–Lemeshow chi-square was 33.75, and the C-statistic (AUC), a measure of the discriminatory power of a predictive model, was 0.69 (95% CI, 0.68 to 0.69). Thus, the explanatory power of the model was 69% (Figure 1).

### Calibration of cancer death prediction in 10 years from cancer survivors

The equation for the cancer mortality prediction model was as follows:

$$\begin{array}{l} \text{KCR}=0.05181\times (\text{AGE}-48.7764)-0.20193\times (\text{GENDER}-1.32291)-\\ 0.00692\times (\text{BMI}-23.211)+0.05613\times (\text{EXSMOK}-0.146)+0.36043\times \\ (\text{CUSMOK}-0.43309)-0.04224\times (\text{EXER}-1.26239)-0.08636\times (\text{FCAN}-\\ 0.26239)-0.08588\times (\text{PHTN}-0.049352)+0.06669\times (\text{PDM}-0.0352);\\ \text{KCR}1=\text{exp}(\text{KCR}); \end{array}$$

BMI, body mass index; EXSMOK, former smoker; CUSMOK, current smoker; EXER, exercise; FCAN, family history of cancer; PHTN, past history of hypertension; PDM, past history of diabetes.

**Figure 2:**
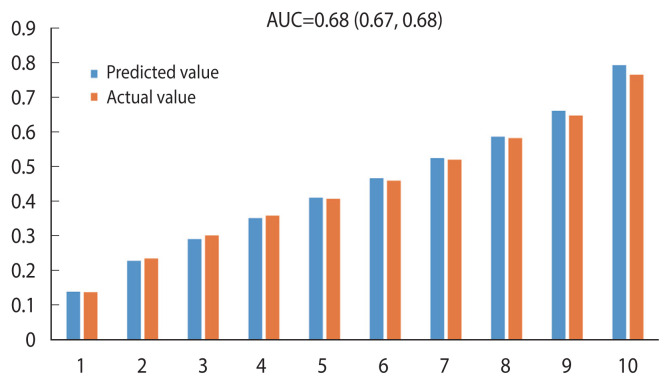
Calibration of 10-year cancer death prediction in cancer survivors. AUC, area under the receiver operating charac teristic curve.

The predicted score for cancer survivors was calculated as 1− 0.58232×KCR1, reflecting the 10-year cancer survival rate. Based on the 10-year cancer mortality prediction model from cancer survival, the Hosmer–Lemeshow chi-square was 66.82, and the C-statistic (AUC) was 0.68 (95% CI, 0.67 to 0.68), suggesting that the explanatory power of the model was 68% (Figure 2).

### Estimated effects of lifestyle modification on mortality risk in cancer survivors

To analyze the effect of modifiable lifestyle factors, the cancer survivors were divided into the following groups: those who maintained their current lifestyle, improved their exercise status, improved smoking status, or improved both exercise status and smoking status.

**Figure 3:**
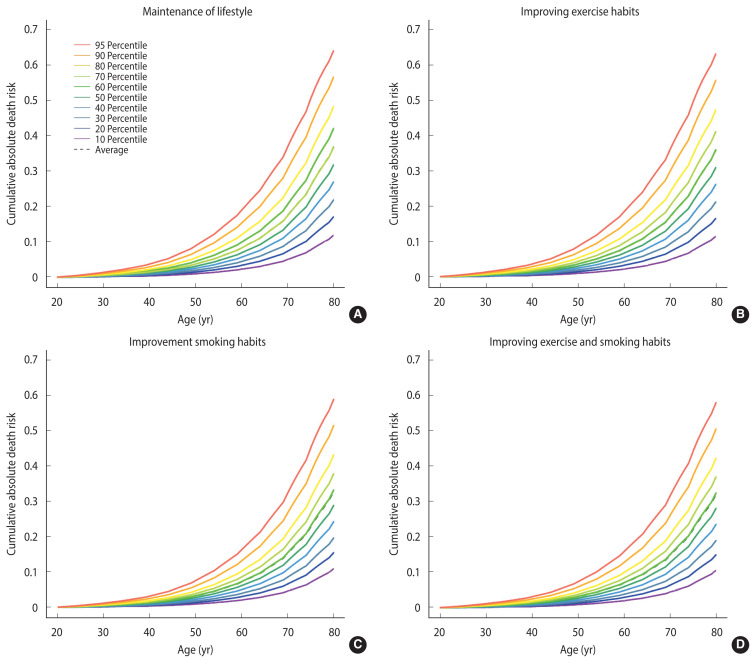
Estimation of the effects of lifestyle modification on cumulative absolute death risk for all-cause mortality among cancer survivors. (A) All cancer survivors maintain exercise and smoking habits, (B) assuming that all cancer survivors exercised, (C) assuming that all cancer survivors did not smoke, and (D) assuming that all cancer survivors exercised and did not smoke.

The risk of all-cause mortality decreased by 1% in cancer survivors who started exercising. Survivors who quit smoking showed a decrease in their risk of death by 5%. In addition, cancer survivors who started exercising and quit smoking showed a decreased risk of death by 6% (Figure 3). There was an equally linear reduction in the risk of cancer death, decreasing from 24% to 20%, in cancer survivors who started exercising. For cancer survivors who quit smoking, the risk of death was reduction to 6%. Additionally, those who started exercising and quit smoking decreased their risk to 3% (Supplementary Material 4).

## DISCUSSION

Our study constructed a model for predicting the death of cancer survivors and examined the effects of lifestyle factors on cancer death and all-cause mortality. The mortality prediction model used lifestyle factors as predictors. Based on the prediction model, we estimated the effect of lifestyle changes on mortality and confirmed that lifestyle modifications reduced the mortality rate.

Of the total 1,637,287 participants, 12.3% (n=200,834) had cancer, and 103,320 died. In our study, the mortality rate of cancer survivors was significantly higher among older men, current smokers, and patients with a history of diabetes. In addition, the lower-BMI group showed a higher mortality rate than the higher-BMI group. In contrast, the mortality rate of cancer survivors was significantly lower in those with a family history of cancer and a history of hypertension.

The prediction model discrimination in the validation dataset (KAR and KCR) was shown by C-statistics of 0.69 and 0.68, respectively. In general, a C-statistic (AUC) of 0.5 suggests no discrimination, 0.7 to 0.8 is considered acceptable, 0.8 to 0.9 is considered excellent, and more than 0.9 is considered outstanding [28]. Based on this information, this study’s models for all-cause mortality prediction and cancer death mortality prediction were both considered close to acceptable. Based on the constructed prediction models, when we changed modifiable factors (exercise status and smoking status), the risk of all-cause mortality and cancer death among cancer survivors was found to decrease linearly.

### Lifestyle factors

Smoking is a known risk factor for cancer, and smoking increases the risk of cancer incidence [5] and mortality. In our study, the all-cancer mortality rate of current smokers was higher than that of non-smokers, and this result was also found in lung, stomach, and colorectal cancers. Several previous studies support this finding [29,30]. In a lung cancer study by Luo et al. [31], smoking cessation after initial primary lung cancer diagnosis could reduce the risk of subsequent malignancy in the lungs. Quitting smoking can reduce the chances of dying from cancer, help cancer treatments work better, and lower the risk of treatment complications. Not only does smoking cessation improve overall health and quality of life, but it also helps cancer survivors feel better and live longer [32]. In our study, those who quit smoking had a risk of death that decreased from 31% to 13%. This result shows the importance of quitting smoking, which was the lifestyle modification associated with the greatest risk reduction.

Exercise is a factor that helps prevent cancer death, as the general population or cancer survivors who engage in a high level of physical exercise show a lower cancer death rate than those who are physically inactive [17]. In an exercise intervention study of breast cancer survivors, the group that received a 16-week combined aerobic and resistance exercise program reported improvements in quality of life, fatigue, depression, muscular strength, osteocalcin, and bone-specific alkaline phosphatase. Based on this result, the researchers suggested that a supervised clinical exercise program should be included in breast cancer care and treatment [33]. Ligibel et al. [34] reported that increased physical activity was associated with both lower breast cancer risk and better outcomes in individuals with early-stage disease. Our study also showed the same result as that of a previous study. The all-cancer mortality rate of those who exercised was lower, and lung, stomach, and colorectal cancers also showed the same results. In contrast, for stomach cancer, the mortality rate of those who exercised was higher than that of those who did not exercise, but the difference was not statistically significant.

Some studies have reported that weight gain after a cancer diagnosis had an adverse effect on mortality in cancer survivors [1,35]. Consistent with prior research [36], this study found that obesity increased the risk of cancer-specific death in individuals with colorectal cancer, but the increase was not statistically significant. However, some of our results are contrary to those of studies showing that higher BMIs increase mortality rates. There was a considerable inverse link between upper aerodigestive tract cancers, including oral, laryngeal, esophageal, and lung cancers, and BMI. Gastric and colorectal cancers were also inversely associated with BMI. Additionally, it has been discovered that chronic lung disease mortality is substantially associated with underweight [37,38]. In East Asian cohorts, which included Chinese, Japanese, and Koreans, those with a BMI between 22,6 and 27,5 had the lowest mortality risk [39]. In a study of Koreans who underwent gastrectomy, underweight patients had a poorer overall survival rate compared to normal-weight patients, but overweight, slightly obese, and moderately obese patients had a greater overall survival rate. Also, disease-specific survival rates followed a similar pattern, with the lowest mortality rates reported among moderately obese adults [40]. According to a previous study, Asian people have lower BMI values, but a higher rate of body fat and different fat distributions than Caucasians [41]. It was also reported that the upper subcutaneous fat percentage is higher in Asians than in Caucasians [42]. Based on these results, our findings are consistent with those of studies reporting that the increased health risk related to obesity occurs at lower BMIs in Asians [43].

The most prevalent comorbidity among cancer survivors is hypertension, and cancer survivors have a higher prevalence of hypertension than the general population [44]. However, cancer survivors are considered to engage in better hypertension management behavior than the general population [45]. Cancer survivors are also more likely to be aware of, treat, and manage high blood pressure, and have better access to knowledge on health-improving behaviors such as vaccination or cancer screening, improvements in nutrition and exercise, and reduction or cessation of smoking and drinking [46,47]. This may explain our finding that a history of hypertension is associated with a decreased mortality rate.

The risk of death in cancer survivors was also found to be lower in those who exercised and had a family history of cancer. A family history of cancer had a protective effect. According to Tracy et al. [48], a family history of breast cancer increases mammography screening, belief in early discovery results, and risk consciousness. In particular, the belief that early discovery can enable the treatment breast cancer at an early stage leads to higher mammography screening rates. Rectal cancer patients who also had family members with rectal cancer showed a higher survival rate and lower risk of recurrence than patients who did not have family members with rectal cancer [49]. Patients with a family history of cancer are a health-conscious group; therefore, early medical examinations can be conducted in these patients, allowing the provision of more abundant health information.

### Strength and limitations

Our study confirmed that the mortality rate decreased when participants’ lifestyles improved. One strength of this cohort study is that it was conducted with a large sample size, a wide age range, and a nationwide sample. Second, it used long-term follow-up data of more than 20 years. Third, this study made a novel contribution by conducting a predictive analysis of death among Asian—and specifically, Korean—cancer survivors. Finally, the model used lifestyle factors as predictors, and the effectiveness of the improvement of lifestyle factors was tested.

Nonetheless, this study has some limitations. First, this study included possible measurement errors, and clinical data from the health promotion centers included one-time measurements of blood pressure and other medical outcomes. Second, the results were likely to be affected by unmeasured and residual confounding factors. Although the analyses were controlled for key demographic indicators, behavioral risk factors, self-reported health, and BMI, the true strength of the association remains uncertain. Third, the C-statistics of 0.68 and 0.69 do not appear impressive. This might be due to limitations of the model when the predictor is insufficient or dichotomized for simplicity [50]. In this study, we only used 8 predictors to identify lifestyle and mortality risk factors and to analyze whether health-promoting lifestyles affect mortality. Fourth, in general, obesity is associated with an increased risk of cancer death; however, our study indicates the contrary. Due to the fact that the risk of obesity may vary by race, the applicability of our findings to other races is limited. Fifth, no familial history information was available for individual cancer types in this study. Lastly, this investigation primarily included individuals of Korean ancestry, potentially limiting the generalizability of the results to individuals of other races or ethnicities. Therefore, in order to generalize the results of this model, further applied research in various populations will be required.

The mortality prediction model for cancer survivors was established using age, gender, BMI, smoking, family history of cancer, exercise, and medical history of hypertension and diabetes. The mortality rate was higher in men, current smokers, and participants with a history of diabetes, whereas the rate was lower in those who exercised and had a family history of cancer. Based on the mortality prediction model, if modifiable lifestyle factors, such as exercise and smoking, can be changed, the cancer-specific and all-cause average mortality rates could be reduced linearly.

In conclusion, the results of this study suggest that the risk of cancer-related mortality can be decreased by lifestyle mortifications.

## Figures and Tables

**Table 1:** PY of follow-up and general characteristics of the Korean Cancer Prevention Study

Characteristics	PY of follow-up	General population	PY of follow-up	Cancer survivors
Total (n)	40,433,731	1,637,287	4,332,402	200,834

Age (yr)		42.9±13.4		48.9±12.0

Systolic blood pressure (mmHg)		122.1±17.2		124.7±18.1

Body mass index (kg/m^2^)		22.9±2.9		23.2±2.9

Gender				
Men	24,309,046	989,744 (60.5)	2,867,569	136,000 (67.7)
Women	16,124,685	647,543 (39.5)	1,464,833	64,834 (32.3)

Age (yr)				
<30	8,241,717	310,284 (19.0)	370,092	14,484 (7.2)
30–39	11,142,966	418,200 (25.5)	762,421	30,882 (15.4)
40–49	9,695,843	377,986 (23.1)	1,177,193	50,762 (25.3)
50–59	782,292	332,136 (20.3)	1,416,419	67,612 (33.7)
60–69	2,805,424	144,009 (8.8)	516,101	30,043 (15.0)
≥70	724,789	54,672 (3.3)	90,175	7,051 (3.5)

Smoking status				
Ex-smoking [men]	4,929,293 [4,707,908]	205,467 (12.6) [11.9]	616,691 [592,695]	29,488 (14.7) [14.0]
Current smoking [men]	14,833,654 [14,431,860]	610,391 (37.3) [36.0]	1,805,641 [1,759,468]	86,992 (43.3) [42.0]

Exercise (yes)	8,885,534	366,718 (22.5)	1,106,514	52,674 (26.4)

Alcohol consumption (yes)	20,570,604	827,268 (50.5)	2,289,975	106,597 (53.1)

Past history of HTN (yes)	1,307,241	57,146 (3.5)	206,173	9,919 (5.0)

Past history of DM (yes)	835,312	38,586 (2.4)	139,606	7,091 (3.5)

Family history of cancer (yes)	4,138,984	166,083 (10.1)	504,774	22,987 (11.5)

Values are presented as mean±standard deviation or number (%).

PY, person-years; HTN, hypertension; DM, diabetes.

**Table 2:** Cancer incidence in the GP and the mortality of cancer survivors

Variables	n (%)	PY of follow-up in GP	Cancer incidence case (CI) in GP	PY of follow-up in CS	All-cause mortality in CS (CDR)	Cancer mortality in CS (CDR)
Total (n)		40,433,731	200,834 (497)	4,332,402	103,320 (2,385)	86,891 (2,006)

Gender						
Men	989,744 (60.5)	24,309,046	136,000 (559)	2,867,569	77,799 (2,713)	65,478 (2,283)
Women	647,543 (39.5)	16,124,685	64,834 (402)	1,464,833	25,521 (1,742)	21,413 (1,462)

Age (yr)						
<30	310,284 (19.0)	8,241,717	14,484 (176)	370,092	2,203 (595)	2,075 (561)
30–39	418,200 (25.5)	11,142,966	30,882 (277)	762,421	8,462 (1,110)	7,827 (1,027)
40–49	377,986 (23.1)	9,695,843	50,762 (524)	1,177,193	19,725 (1,676)	17,734 (1,506)
50–59	332,136 (20.3)	782,292	67,612 (8,643)	1,416,419	40,904 (2,888)	34,210 (2,415)
60–69	144,009 (8.8)	2,805,424	30,043 (1,071)	516,101	25,271 (4,897)	19,933 (3,862)
≥70	54,672 (3.3)	724,789	7,051 (973)	90,175	6,755 (7,491)	5,112 (5,669)

GP, general population; PY, person-years; CI, cumulative incidence per 100,000 PY; CS, cancer survivors; CDR, crude death rate per 100,000 PY.

**Table 3:** Deaths among cancer survivors by causes

Cancer	ICD-code	Person-years of follow-up	Cancer incidence	All-cause death (CDR)	All-cancer death (CDR)	Cancer-specific death (CDR)	Others, n (%)
Stomach cancer	C16	871,485	39,926	19,177 (4,581)	15,063 (1,728)	12,419 (1,425)	4,114 (21.5)
Colorectal cancer	C18–C20	649,524	28,527	13,075 (4,392)	10,436 (1,607)	8,564 (1,319)	2,639 (14.6)
Lung cancer	C34	488,971	26,711	21,498 (5,463)	19,705 (4,030)	18,024 (3,686)	1,793 (8.3)
Liver cancer	C22	402,635	22,941	18,460 (5,698)	16,812 (4,175)	13,954 (3,466)	1,648 (8.9)
Thyroid cancer	C73	490,726	18,602	1,170 (3,791)	784 (160)	362 (74)	386 (33.0)
Prostate cancer	C61	316,913	12,798	4,251 (4,038)	2,843 (897)	2,062 (651)	1,408 (33.1)
Breast cancer	C50	287,451	11,388	1,985 (3,962)	1,685 (586)	1,426 (496)	300 (15.1)
Gallbladder cancer	C23, C24	138,386	7,685	6,315 (5,553)	5,822 (4,207)	4,122 (2,979)	493 (7.8)
Pancreas cancer	C25	119,445	6,869	6,256 (5,751)	5,923 (4,959)	5,231 (4,379)	333 (5.3)
Bladder cancer	C67	154,689	6,677	3,079 (4,316)	2,191 (1,416)	1,398 (904)	888 (28.8)
Esophageal cancer	C15	61,703	3,395	2,665 (5,502)	2,412 (3,909)	1,969 (3,191)	253 (9.5)
Oral cancer	C00–C14	68,044	3,242	1,942 (4,765)	1,626 (2,390)	1,058 (1,555)	316 (16.3)
Brain cancer	C71	42,842	2,334	1,870 (5,448)	1,537 (3,588)	1,059 (2,472)	333 (17.8)
Cervical cancer	C53	51,814	2,297	855 (4,433)	680 (1,312)	447 (863)	175 (20.5)
Laryngopharyngeal cancer	C32	39,382	1,833	1,039 (4,654)	784 (1,991)	422 (1,072)	255 (24.5)

ICD, International Classification of Diseases; CDR, crude death rate per 100,000 person-years.

**Table 4:** Risk factors for cancer mortality in cancer survivors^1^

Variables	All cancer	Lung cancer	Stomach cancer	Colorectal cancer
Age (yr)	1.05 (1.05, 1. 05)	1.07 (1.07, 1.07)	1.06 (1.06, 1.06)	1.06 (1.06, 1.07)
Gender (women)	0.83 (0.81, 0.84)	0.91 (0.86, 0.95)	0.70 (0.67, 0.75)	0.87 (0.81, 0.92)
BMI (≥23.0 kg/m^2^)	0.99 (0.99, 0.99)	0.95 (0.95, 0.96)	0.97 (0.97, 0.98)	1.01 (1.01, 1.02)
Smoking (ex-smoking)	1.06 (1.04, 1.09)	1.30 (1.22, 1.39)	1.03 (0.96, 1.09)	1.03 (0.96, 1.10)
Smoking (current smoking)	1.44 (1.42, 1.47)	3.15 (3.00, 3.31)	1.32 (1.25, 1.39)	1.05 (0.98, 1.10)
Family history of cancer (yes)	0.93 (0.91, 0.95)	0.91 (0.87, 0.95)	0.97 (0.92, 1.02)	0.88 (0.82, 0.95)
Exercise (yes)	0.97 (0.96, 0.98)	0.92 (0.89, 0.95)	1.01 (0.97, 1.05)	0.94 (0.90, 0.99)
Past history of HTN (yes)	0.93 (0.90, 0.96)	0.94 (0.88, 1.01)	0.86 (0.79, 0.93)	0.98 (0.90, 1.07)
Past history of DM (yes)	1.09 (1.06, 1.13)	0.95 (0.88, 1.03)	1.03 (0.94, 1.13)	1.04 (0.93, 1.15)

Values are presented as hazard ratio (95% confidence interval).

BMI, body mass index; HTN, hypertension; DM, diabetes.

1 Adjusted for age, gender, BMI, smoking, family history of cancer, exercise, past history of HTN, and past history of DM.

## SUPPLEMENTARY MATERIALS

Supplementary Material 1. Flowchart of the study population

Supplementary Material 2. Characteristics of the derivation and validation cohorts in cancer survivor

Supplementary Material 3. The risk factors for cancer mortality in cancer survivors

Supplementary Material 4. Effect estimation through lifestyle modification of cancer death from cancer survivors
