# Supplementary material for: Prediction of cancer survivors’ mortality risk in Korea: a 25-year nationwide prospective cohort study
Source: Epidemiol Health. 2022 Sep 13;44:e2022075. doi: 10.4178/epih.e2022075 (PMC9943637; doi:10.4178/epih.e2022075)
Supplement: Supplementary Material 1. — Flowchart of the study population [file epih-44-e2022075-Supplementary-1.docx]

**Supplementary Material 1. Flowchart of the study population**


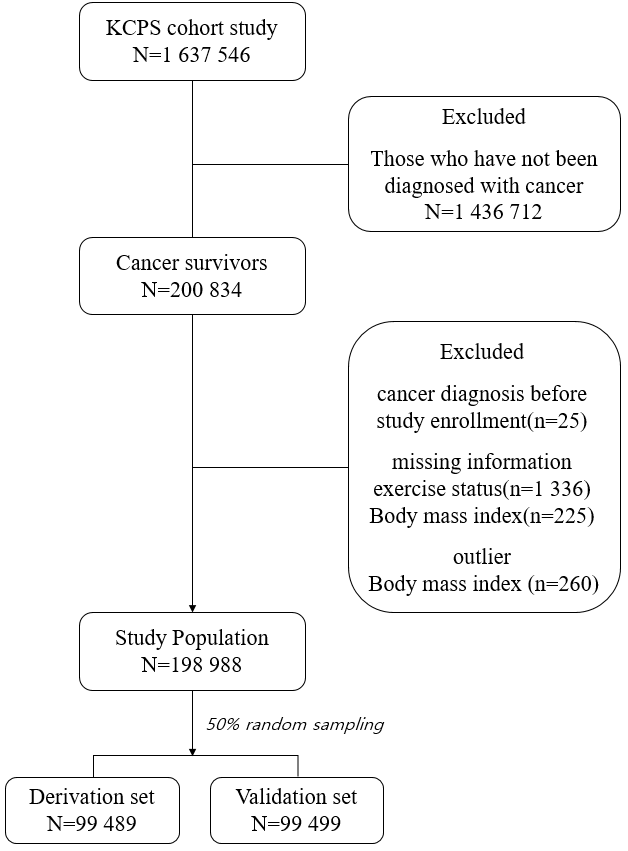


- BMI outlier is an extremely high (>100 kg/m2) or low BMI (<16 kg/m2)
