# Supplementary material for: Prediction of cancer survivors’ mortality risk in Korea: a 25-year nationwide prospective cohort study
Source: Epidemiol Health. 2022 Sep 13;44:e2022075. doi: 10.4178/epih.e2022075 (PMC9943637; doi:10.4178/epih.e2022075)
Supplement: Supplementary Material 2. — Characteristics of the derivation and validation cohorts in cancer survivor [file epih-44-e2022075-Supplementary-2.docx]

**Supplement Material 2. Characteristics of the derivation and validation cohorts in cancer survivor**

|  |  |  | N, (%) |
| --- | --- | --- | --- |
|  | **Derivation** |  | **Validation** |
| **Characteristic (n=198,988)** | **(n=99,489)** |  | **(n=99,499)** |
| Gender |  |  |  |
| Men | 67,363(67.7) |  | 67,469(67.8) |
| Women | 32,126(32.3) |  | 32,030(32.2) |
| Age, mean(SD), y | 48.78(12.0) |  | 48.81(12.0) |
| Age, group, y |  |  |  |
| 20-30 | 7,268(7.3) |  | 7,156(7.2) |
| 30-39 | 15,464(15.5) |  | 15,347(15.4) |
| 40-49 | 25,257(25.4) |  | 25,323(25.5) |
| 50-59 | 33,371(33.5) |  | 33,554(33.7) |
| 60-69 | 14,647(14.7) |  | 14,745(14.8) |
| 70+ | 3,482(3.5) |  | 3,374(3.4) |
| BMI, mean(SD) | 23.2(2.8) |  | 23.2(2.8) |
| BMI |  |  |  |
| obese (25kg/m^2^+) | 24,979(25.1) |  | 25,020(25.2) |
| over weight (23kg-24.9kg/m^2^) | 26,116(26.3) |  | 26,236(26.4) |
| normal (18.5kg-22.9kg/m^2^) | 44,951(45.2) |  | 44,819(45.0) |
| under weight (18.5kg/m^2^-) | 3,443(3.5) |  | 3,424(3.4) |
| Smoke status |  |  |  |
| non-smoker | 41,876(42.1) |  | 41,703(41.9) |
| former smoker | 14,525(14.6) |  | 14,726(14.8) |
| current smoker | 43,088(43.3) |  | 43,070(43.3) |
| Family history of cancer |  |  |  |
| Yes | 11,396(11.5) |  | 11,442(11.5) |
| No | 88,093(88.6) |  | 88,057(88.5) |
| Exercise |  |  |  |
| Yes | 26,105(26.4) |  | 26,457(26.6) |
| No | 73,384(73.8) |  | 73,042(73.4) |
| Past history of HTN |  |  |  |
| Yes | 4,910(4.9) |  | 4,974(5.0) |
| No | 94,579(95.1) |  | 94,525(95.0) |
| Past history of DM |  |  |  |
| Yes | 3,502(3.5) |  | 3,558(3.6) |
| No | 95,987(96.5) |  | 95,941(96.4) |
