# Supplementary material for: Prediction of cancer survivors’ mortality risk in Korea: a 25-year nationwide prospective cohort study
Source: Epidemiol Health. 2022 Sep 13;44:e2022075. doi: 10.4178/epih.e2022075 (PMC9943637; doi:10.4178/epih.e2022075)
Supplement: Supplementary Material 3. — The risk factors for cancer mortality in cancer survivors [file epih-44-e2022075-Supplementary-3.docx]

**Supplement Material 3. The risk factors for cancer mortality in cancer survivors**

|  | **All cancer**  (85,812/198,988) | **Lung cancer**  (18,568/198,988) | **Stomach cancer**  (12,621/198,988) | **Colorectal cancer**  (8,674/198,988) |
| --- | --- | --- | --- | --- |
|  | Hazard Ratio (95% CI) | | | |
| Age (year) | 1.05(1.05-1.05) | 1.07(1.07-1.07) | 1.06(1.06-1.06) | 1.06(1.06-1.07) |
| Gender (women) | 0.82(0.80-0.84) | 0.90(0.85-0.95) | 0.70(0.66-0.74) | 0.87(0.81-0.92) |
| BMI (18.5kg/m^2^-) | Ref. | Ref. | Ref. | Ref. |
| BMI (18.5kg-22.9kg/m^2^) | 0.90(0.87-0.94) | 0.81(0.75-0.87) | 0.79(0.73-0.87) | 1.01(0.89-1.15) |
| BMI (23kg-24.9kg/m^2^) | 0.85(0.82-0.89) | 0.66(0.61-0.71) | 0.67(0.61-0.74) | 1.04(0.91-1.18) |
| BMI (25kg/m^2^+) | 0.88(0.85-0.92) | 0.62(0.57-0.67) | 0.72(0.65-0.79) | 1.10(0.97-1.26) |
| Smoking -Ex | 1.06(1.04-1.09) | 1.30(1.23-1.39) | 1.03(0.96-1.09) | 1.03(0.95-1.10) |
| Smoking –Current | 1.44(1.41-1.47) | 3.16(3.01-3.32) | 1.32(1.25-1.39) | 1.04(0.98-1.11) |
| Family history of cancer (Y) | 0.93(0.91-0.95) | 0.91(0.86-0.95) | 0.97(0.92-1.02) | 0.88(0.82-0.95) |
| Exercise (Y) | 0.97(0.96-0.99) | 0.92(0.89-0.95) | 1.02(0.98-1.06) | 0.94(0.90-0.99) |
| Past history of HTN (Y) | 0.93(0.90-0.96) | 0.93(0.87-0.99) | 0.85(0.79-0.92) | 0.98(0.90-1.07) |
| Past history of DM (Y) | 1.09(1.06-1.13) | 0.95(0.88-1.03) | 1.03(0.94-1.13) | 1.04(0.93-1.15) |

Adjusted for age, sex, body mass index, smoking, family history of cancer, exercise, history of hypertension, and history of DM

Abbreviations: BMI, body mass index; HTN, hypertension; DM, diabetes; HR, hazard ratio; CI, confidence interval
