# Supplementary material for: Prediction of cancer survivors’ mortality risk in Korea: a 25-year nationwide prospective cohort study
Source: Epidemiol Health. 2022 Sep 13;44:e2022075. doi: 10.4178/epih.e2022075 (PMC9943637; doi:10.4178/epih.e2022075)
Supplement: Supplementary Material 4. — Effect estimation through lifestyle modification of cancer death from cancer survivors [file epih-44-e2022075-Supplementary-4.docx]

**Supplementary Material 4. Effect estimation through lifestyle modification of cancer death from cancer survivors**

Hazar ratio
